# Regulation of root growth and elongation in wheat

**DOI:** 10.3389/fpls.2024.1397337

**Published:** 2024-05-21

**Authors:** Abdullah Alrajhi, Saif Alharbi, Simon Beecham, Fahad Alotaibi

**Affiliations:** ^1^ King Abdulaziz City for Science and Technology (KACST), Riyadh, Saudi Arabia; ^2^ Sustainable Infrastructure and Resource Management, University of South Australia, University of South Australia Science, Technology, Engineering, and Mathematics (UniSA STEM), Mawson Lakes, SA, Australia; ^3^ The National Research and Development Center for Sustainable Agriculture (Estidamah), Riyadh, Saudi Arabia

**Keywords:** wheat, root elongation, phenotyping, molecular genetics, root development, root growth

## Abstract

Currently, the control of rhizosphere selection on farms has been applied to achieve enhancements in phenotype, extending from improvements in single root characteristics to the dynamic nature of entire crop systems. Several specific signals, regulatory elements, and mechanisms that regulate the initiation, morphogenesis, and growth of new lateral or adventitious root species have been identified, but much more work remains. Today, phenotyping technology drives the development of root traits. Available models for simulation can support all phenotyping decisions (root trait improvement). The detection and use of markers for quantitative trait loci (QTLs) are effective for enhancing selection efficiency and increasing reproductive genetic gains. Furthermore, QTLs may help wheat breeders select the appropriate roots for efficient nutrient acquisition. Single-nucleotide polymorphisms (SNPs) or alignment of sequences can only be helpful when they are associated with phenotypic variation for root development and elongation. Here, we focus on major root development processes and detail important new insights recently generated regarding the wheat genome. The first part of this review paper discusses the root morphology, apical meristem, transcriptional control, auxin distribution, phenotyping of the root system, and simulation models. In the second part, the molecular genetics of the wheat root system, SNPs, TFs, and QTLs related to root development as well as genome editing (GE) techniques for the improvement of root traits in wheat are discussed. Finally, we address the effect of omics strategies on root biomass production and summarize existing knowledge of the main molecular mechanisms involved in wheat root development and elongation.

## Introduction

Wheat (*Triticum aestivum* L.) is the world’s most widely cultivated grain crop, covering some 219 Mha of land (Singh et al., 2024), and serving as a staple food for humans. It accounts for 18% of people’s daily calorie intake and 200-% of their daily protein. The world demand for wheat is expected to increase by 60% by 2050 due to the increasing global population (Leegood et al., 2010). The current wheat yield will have to increase by 1.7% per year to meet this increased demand. Achieving this objective is a major challenge under current climate change scenarios, as models predict a 25–30% decrease in precipitation and a 4–5°C increase in temperature for the Mediterranean region (Giorgi and Lionello, 2008), which would adversely affect wheat cultivation. Wheat output is well known to be heavily influenced by ecological stresses such as salinity, drought, and heat (Uga et al., 2015). Underground roots play a significant role in plant growth because they absorb the water and nutrients necessary for developmental processes (Sharma et al., 2009). Consequently, specific traits for breeding toward a particular root system architecture (RSA) provide better tolerance by developing specific wheat genotypes that survive under harsh conditions, resulting in improved production (Sanguineti et al., 2007). The water and nutrients available for photosynthesis are determined by root systems, which influence the characteristics and yield of harvested products and therefore underlie agricultural productivity (Tracy et al., 2020). A narrow root growth angle encourages deeper root development and is commonly linked to enhance the access to water and nutrients in deep soils, particularly during periods of terminal drought. The bulk of the mature root system in cereal crops is produced by several types of adventitious roots, including crown roots and brace roots. In particular, the RSA, which refers to the spatial arrangement of soil roots, shows significant plasticity due to the heterogeneous distribution of soil capital and terrestrial variations (Abelson, 1999). Wheat root morphology and/or anatomical characteristics help the plant to sustain higher grain yields with low availability of resources, such as a relatively deeper root distribution that increases water consumption during droughts (Reynolds et al., 2007).

Since evaluating RSA in the field is problematic, as well as costly and laborious when phenotyping for many genotypes, several studies have been conducted to screen RSA characteristics at the initial growth level (Sanguineti et al., 2007). Among various RSA characteristics, root length, root numbers, area covered, volume, and root diameter are associated with the root structure and the absorption of nutrients and water, and therefore also with tolerance mechanisms. Seminal root angle (SRA) is another significant feature that is used to determine the RSA; a narrow angle may lead to vertical movement of root growth to obtain moisture from deeper soil layers and thus sustain a higher yield (Tracy et al., 2020). Modern industrialized farming has become dependent on fertilizers, which poses risks of acute limitation of the components in non-renewable fertilizers, especially phosphorus (P) (Cordell et al., 2009). Certain nutrients, such as P, zinc, and iron, are absorbed by plants at the root-soil interface, so plants frequently face conditions under which one or more of these elements are restricted (Cane et al., 2014). In addition, challenges are being faced in enhancing a plant’s ability to absorb these nutrients from the soil for sustainable production and growth. Some studies show that the conserved signaling target for rapamycin (*TOR*) is essential for controlling the root growth in drought conditions (Xiong and Sheen, 2014). The *TOR* kinase is a crucial regulator of animal and yeast cell growth and differentiation and appears to play a central role in regulating plant environmental and hormonal responses (Barrada et al., 2015).

Current phenotyping techniques are expected to expedite the development of desired root traits, which can be combined in parallel with several new germplasm alleles for target environments (Maccaferri et al., 2016). QTLs are identified in segregating populations or panels of genotypes through genome wide association study (GWAS). Once a QTL has been detected, it can be introgressed or manipulated through marker-assisted selection (MAS). GWAS have recently gained popularity through germplasm collections, which offer wider variation than that achieved through traditional breeding crosses. Such collections facilitate the identification of multiple recombination events, leading to precise characterization of relationships between phenotype and genotype. Landrace assemblies are essential for GWAS (Hochholdinger and Tuberosa, 2009) as they represent genetically diverse collections with unique characteristics developed under local growing conditions, encompassing a wide range of biotic and abiotic stresses. Mediterranean wheat genotypes have demonstrated a robust genetic history attributed to their root structure and function, yield, quality, and stress tolerance (Xynias et al., 2020). Molecular breeding techniques have focused on genes that regulate RSA; by optimizing soil moisture uptake and retention, particularly under drought condition, aiming to enhance root structure.

Contemporary advances in the field of genomics and reverse genetics techniques in wheat, together with the development of reference genome sequence data and the introduction of GE technologies, are all necessary to decode the functional pathways, genes and their function related to regulatory networks based on phenological adaptation characteristics. These tools lay the groundwork for elucidating further details of this complex molecular interaction, enabling the control of phenotypic differences and the identification of new mechanisms involved in the root development and elongation processes. These recent integrated approaches have underscored the intricate development processes necessary for establishing biological and architectural foundations. Therefore, the molecular procedures that underpin the modification of root growth have been instrumental in facilitating the understanding of simple principles. Despite the significance of roots, they have largely been neglected in modern crop research and breeding efforts (Voss-Fels et al., 2018a, b). Here, we delve into significant advancements in crop root research and underscore how the context-dependent optimization of both underground and aboveground plant components presents opportunities to enhance future crops amidst escalating environmental fluctuations. The remainder of this paper is structured as follows: The first part discusses RSA, transcriptional control of apical meristem and root elongation, root architecture and hormone distribution, phenotyping of the root system, and simulation models that control the root functions and elongation process. In the second part, we discuss the molecular genetics of the root system, with respect to single nucleotide polymorphism (SNP), transcription factors (TFs), and QTLs related to root development, as well as the GE technique for improving root traits in wheat.

## Root system architecture

**Figure 1:**
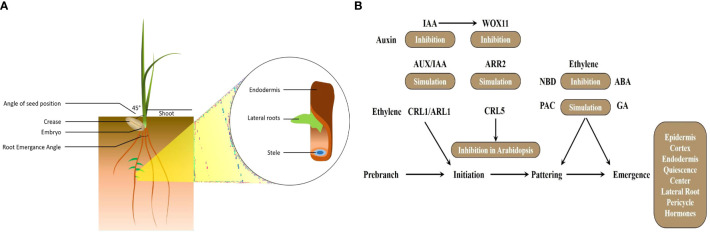
**(A)** Wheat root system architecture. **(B)** Gene regulatory pathway involved in adventitious roots in cereals. NBD, 2,5-norbornadiene (ethylene inhibitor); ARR, Arabidopsis response regulator; PAC, paclobutrazol (GA inhibitor); WOX, WUSCHEL-related homeobox; Aux/IAA, auxin/indole acetic acid.

The wheat primary root comprises three distinct zones: meristematic, elongation, and maturation, each delineating specific stages of cell growth and development, offering valuable insights into cell growth regulation. Root morphology, including features like root hairs, cortical senescence, and root diameter, outlines key characteristics of a single root axis (**Figure 1A**). Understanding the RSA and morphology is crucial for optimizing crop yield. Izzi et al. (2008) studied wheat root growth and soil water extraction in a Mediterranean climate, highlighting peak root growth during and before the late irrigation season. Liu et al. (2004) observed that extreme water shortages significantly increase root respiration and water intake needs in drought-sensitive wheat in arid regions. In monocots i.e., maize, wheat, and rice, the root system comprises main, seminal embryonic roots (SRs), shoot-borne roots, and postembryonic lateral roots (LRs) (Shekhar et al., 2019). Wheat’s root system, characterized by SRs and secondary roots, plays a vital role in drought adaptation (Sinha et al., 2018). RSA features, such as height, surface area, volume, angle, number, and diameter are critical for wheat’s adaptation to drought and nutritional limitation in the root zone area (Maqbool et al., 2022). However, accurately phenotyping roots under various environmental conditions remains challenging due to their complexity and subterranean location (Motte et al., 2019). Phytohormones, light, and nutrient signals regulate root development, coordinating proliferation and elongation processes, although the exact mechanisms remain incompletely understood (Singh et al., 2023). Vigorous RSA features contribute to high grain iron (Fe) concentration among wheat germplasms (Sultana et al., 2023). This could be attributed to the influence of genotype and environmental conditions, which affect the phytoavailable mineral content in wheat (Ishfaq et al., 2021).

Plant meristem stem cells play diverse roles in the growth and expansion of new plant organs. The shoot apical meristem (SAM) produces aboveground organs, while the root apical meristem (RAM) generates underground organs (Semenova, 2017). In monocots like wheat, most seedling components originate from SAMs, as the radicle system primarily consists of adventitious roots. Environmental cues can influence RAM formation, and any deviations from the typical development program can impact plant survival (Song et al., 2023). In attempting to correlate root elongation with cellular processes, Shimazaki et al. (2005) concluded that inadequate water supply in the apical meristem zone could result in a significant reduction in maize root growth. Stress can disrupt the RAM establishment, potentially leading to programmed cell death in RAM cells under severe osmotic stress. Terletskaya et al. (2020) suggested significant plasmolysis development in the meristematic cells of primary roots in both *T. monococcum* and *T. aestivum*. Additionally, *T. aestivum* exhibited strong plasmolysis in root hair cells, crucial for enlarging the root surface area and enhancing water and soil solution absorption into the root system. The number of pericycle cell files involved in lateral root primordium development varies between species; wheat typically has four to six files, while Arabidopsis has six to eight (Tsukagoshi et al., 2010; Xin, 2023). Understanding these intricate processes sheds light on root development and offers insights into wheat plant adaptation and survival strategies under harsh environmental condition.

## Transcriptional control of apical meristem and root elongation

Most of the time, complex mechanisms are involved in the RAM dynamics, and these tightly control the process by which stem cells differentiate. Two short-root (*SHR*) and *SCARECROW* (*SCR*) homologues have been described for the monocot rice with differing number of cortical tissue layers and morphologies. These homologues may be identical to those found in Arabidopsis regarding cortex and endodermis specification (Nawy et al., 2005). In waterlogging and associated stresses condition, a host of anaerobic reactors and enzymes linked to fermentation pathways are involved, such as alcohol dehydrogenase, pyruvate decarboxylase and other TFs (Fukao and Bailey-Serres, 2004). A William tumor gene 1 (*Wt1)* is necessary to achieve a reasonable level of water-logging resistance when sensitive and tolerant spring bread wheat lines are crossed in a homozygous-dominant state, along with one of the three other genes (*Wt2/wt2, Wt3/wt3*, and *Wt4/wt4*) (Boru et al., 2001).

Screening of Arabidopsis seedlings with a *Ds-GUS* transposon gene-trap function for low oxygen level-induced genes (Baxter-Burrell et al., 2003) revealed that Rho of plants protein signaling was involved in the reaction to hypoxia (Smokvarska et al., 2021). Therefore, it is essential to characterize the changes in transcript expression for different genes associated with decreases in the concentration of oxygen, oxygen signaling during hypoxia, and upstream genes. The *MYB77-*TF was found to be differentially expressed in waterlogged oxygen (low oxygen) roots. The first isolated TF was the plant *MYB* TF, like mammalian TF *c-MYB* (Xu et al., 2006). The *MYB-TF* was highly expressed under low oxygen in roots. The *TaMyb1* root expression may be closely linked to root oxygen levels and abiotic stress responses of the wheat plant (Lee et al., 2007). Sarkar et al. (2007) reported that *WUSCHEL-LIKE HOMEOBOX* (WOX) and *WUSCHEL* (WUS) genes maintained the signals for stem cells in root or shoot cell. *WOX5* specifically promotes cell proliferation by suppressing *CYCD3;3* expressions (Zhang et al., 2017). *WOX5* homologues found in other dicots and anciently diverged lines functionally complement the *WOX5* mutant (Vailleau et al., 2002), indicating that *WOX5* has a broadly maintained role in the niche of stem cells. Recent studies have shed light on the intricate mechanisms governing root architecture and plant development in cereal crops, providing valuable insights for agricultural improvement (Calleja-Cabrera et al., 2020).

Fusi et al. (2022) elucidated the crucial role of enhanced gravitropism1 (*EGT1)* in regulating root angle by modulating cell wall stiffness in elongating root cortical tissue. This anti-gravitropic mechanism counters the natural tendency of roots to bend, presenting a potential target for enhancing root angle control in cereals. Similarly, research by Kirschner et al. (2021) focused on *EGT2* orthologs in tetraploid durum wheat, revealing their involvement in regulating root growth angle across cereal species. RNA-Seq analysis identified seven down-regulated genes in the elongation zone, further confirming *EGT2’s* role in root growth regulation and suggesting tailored strategies for crop improvement. Investigations into the ERF family, as outlined by Zhuang et al. (2021), highlighted their significance in plant development and stress response. Particularly, the study revealed short root length1 (*TaSRL1’s*) impact on root architecture modulation through its influence on auxin-related genes, offering potential implications for crop breeding programs. Furthermore, Gabay et al. (2023) explored the role of genes from subfamily III represents 12-OXOPHYTODIENOATE REDUCTASE (*OPRIII*) in wheat root structure under water-limited conditions. Loss-of-function mutations in these genes resulted in longer seminal roots, while increased expression led to altered lateral root growth patterns, emphasizing their importance in adapting wheat to environmental stresses.

However, TFs associated with MYB in wheat have not been thoroughly characterized to date. Churin et al. (2003) proposed comprehensive sequences and connections between the promoters of chlorophyll a/b-binding protein (CAB) and MYB-like TFs, which were subsequently identified in wheat and barley. The PLETHORA (*PLT*) family is involved in the master root control system (Li L. et al., 2019). Auxin accumulation induces the expression of *PLT* genes. This feedback mechanism helps, to some extent, in further customizing the *PLT* domain in the stem cell niche. The stability of the *PLT* protein depends heavily on peptide signaling of the sulfated root growth factor *(RGF)/GOLVEN* (GLV). Exogenous application of *RGF/GLV* allows the RAM to extend (Whitford et al., 2012) compared to plants that overexpress *PLT*. In Arabidopsis, PLT genes have been demonstrated to act as dose-dependent master regulators of root development (Galinha et al., 2007), however, in rice, these genes are expressed within the stem cell niche of the root and in the emerging lateral roots (Li and Xue, 2011). In wheat *PLT1*, *PLT3*, and *PLT5* gene expression in lines with heightened *OPRIII* dosage or expression may have played a role in halting root meristem growth and/or altering the lateral root distribution (Gabay et al., 2023). Li et al. (2016) identified a plant-specific TF, known as MORE ROOT (*TaMOR*), and demonstrated that overexpressing the wheat *TaMOR* gene in rice (*Oryza sativa*) leads to a larger root system and increased grain yield. Overexpressing a wheat NAC-TF gene promotes root growth and enhances plant drought tolerance in wheat. Interestingly, *TaTRIP1*, a negative regulator in brassinosteroids signaling, has been found to be up-regulated in short-root wheat varieties (He et al., 2014). The variation in ROS accumulation between the LR and SR groups could result in distinct meristem activity, thereby influencing both primary root length (PRL) and maximum root length (MRL). Brassinosteroids (BRs) serve as crucial regulators in numerous biological processes (Haubrick and Assmann, 2006). Furthermore, MAPK cascades play a role in regulating ROS production during BR-mediated responses (Xia et al., 2009). Overexpressing *TaTRIP1*, a negative regulatory gene of wheat BR signaling, in Arabidopsis results in reduced root meristem size and consequently PRL (He et al., 2014).

### Root architecture and hormone distribution

The formation of lateral cereal roots progresses through three developmental stages: organ initiation, cortex growth, and epidermis emergence (Dhaka et al., 2017). Unlike Arabidopsis, maize and wheat exhibit a complex cortical structure with multiple layers surrounding the vascular poles (Dhaka et al., 2017). Lateral roots in cereal plants, including maize, wheat, rice, and barley, originate from pericycle and endodermal cells, differing from eudicots (Hochholdinger and Tuberosa, 2009; Muthreich et al., 2013). The development of lateral roots in cereals involves cell division, hydrolytic enzymes, and hydrogen peroxide-mediated cell death (Casimiro et al., 2003; Draye et al., 2010; Zhou et al., 2010). Additionally, variations in auxin-related proteins influence root initiation and vascular patterning in cereals (Casimiro et al., 2003; Orman-Ligeza, 2015). Cereal plants possess various types of adventitious roots, including crown roots and brace roots, which develop at nodes (Ashraf et al., 2019). Crown roots, found underground, and brace roots, aboveground, are crucial for monocot species lacking seminal and main roots (Liu et al., 2020). The initiation of lateral roots in wheat involves cell proliferation in various root tissues, including pericycles and endodermis, before connecting to the vascular bundle network (Viana et al., 2022). Auxin plays a vital role in regulating root formation, with its transportation controlled by crown rootless4 (*CRL4*) (Cho et al., 2014). In wheat, on the proximal side of the elongated region, at 15–20 mm from the root tip, the first morphological events of lateral root initiation are observed (Placido et al., 2020). In maize, the auxin/IAA protein RUM1 (Rootless with undetectable rootless meristems 1), encoding auxin/IAA, contributes to root initiation failure in lateral roots and disruption of vascular patterning (Ravazzolo, 2019). Variations at the C-terminus, as well as gain and loss of AuxREs and other cis-regulatory elements, potentially play a role in the evolution of deeper rooting 1 (*DRO1*)-like paralogs involved in deeper rooting in wheat (Khan et al., 2017).

The upregulation of auxin and cytokinin signals is facilitated by their promoter cytokinin oxidase. *OsCKX4* is a direct binding targets of both the auxin response factors *OsARF25*, and the cytokinin response regulators *OsRR2* in rice. These signals are predominantly expressed at the base of the trunk, where the crown roots initiate, due to the abundance of cytokinin (Gao et al., 2014). Interactions with other hormones, such as ethylene, gibberellic acid (GA) and abscisic acid (ABA) influence the later phases of crown root growth (Steffens and Rasmussen, 2016). In a wide range of monocots, brace roots develop adventitious roots from aboveground nodes (Wang et al., 1994). Two allelic mutants i.e., *rtcs-1* and *rtcs-2*, are root deficient (adventitious and seminal lateral roots), and survive with the ability of primary root, suggesting genetically programmed and/or hormonally controlled pathways of adventitious root in maize. The monogenic and recessive mutants appear to be affected in early root forming functions (Hetz et al., 1996). Chen et al. (1998) unveiled the intricate interplay of multiple hormones in plants, crucially involving auxin, to regulate root biomass production. Auxin serves as a fundamental signaling molecule, influencing embryo root growth during embryogenesis, and guiding subsequent root meristem organization in response to various stimuli controlling the root growth and developmental process. Furthermore, Tanimoto (2005) suggested the involvement of auxin-responsive gene repressor proteins in root development, while Alvey and Harberd (2005) proposed Ca^2+^-mediated signals inducing hydrolytic enzymes like α-amylase in cereal aleurone cells. Notably Gilroy (1996), proposed that GA increases the levels of Ca^2+^ and calmodulin in barley aleurone cells, but ABA counteracts the effect of GA, and findings indicated that GA and ABA signals are transduced by both Ca2+-dependent and Ca2+-independent systems involving calmodulin (CaM) in the aleurone cell emphasizing hormonal regulation’s complexity in root physiology. Another study reported serine/threonine protein kinase, *PKABA1* mRNA, induced by ABA in aleurone layers of barley. The results suggested that *PKABA1* serves as an important intermediary in the signal transduction cascade pathway responsible for suppressing GA-inducible gene expression in aleurone layers (Gomez-Cadenas et al., 1999). The role of *DRO1* gene and its orthologs, particularly in response to drought stress, has been elucidated by Ashraf et al. (2019) and suggested that the differential expression patterns of *TaBDRO1* TF in wheat roots, promote root development under drought stress condition. Additionally, Barbez et al. (2017) suggested that endogenous auxin regulates apo plastic acidification and the initiation of cellular elongation in roots. Conversely, either an endogenous or exogenous rise in auxin levels triggers a temporary alkalization of the extracellular matrix, leading to reduced cellular elongation in Arabidopsis. This complexity extends to root elongation regulation, where low auxin concentrations modulate root growth, as observed in Arabidopsis. Zhou et al. (2020) highlighted the role of reactive oxygen species (ROS) signaling in balancing cell proliferation and elongation in root development in Arabidopsis. Furthermore, Kitomi et al. (2008) identified mutations *CRL4* in Arabidopsis that was observed to exhibit abnormal crown root formation. In the *CRL4* mutant, both shoot and root auxin transport were impaired. The results suggested that the accumulation and gradient of auxin through *CRL4* in the basal part of shoots are crucial for crown root formation in rice.

Similarly, the phytohormone ethylene is recognized as its function in LR formation, yet the specific factors regulating ethylene during this process remain unclear. Wheat *TaWRKY51* plays a crucial role in LR formation by regulating ethylene biosynthesis. Wheat lines with RNA interference targeting *TaWRKY51* (TaWRKY51-RNAi) and homozygous mutants *TAWRKY51-2a* and *TAWRKY51-2b* exhibited reduced LR production compared to wild-type and negative transgenic plants. Conversely, *TaWRKY51* overexpression lines (*TaWRKY51-OE*) displayed an opposite phenotype with increased LR formation (Hu et al., 2018). Ethylene significantly influences root development, with its function tightly intertwined with auxin regulation. For instance, the application of ethylene leads to the accumulation of auxin in the root tips, a process mediated by WEAK ETHYLENE INSENSIVE2/ANTHRANILATE SYNTHASE α1 (*WEI2/ASA1*) and WEI7/INSENSIVE2/ANTHRANILATE SYNTHASE β1 (*WEI7/ASB1*) (Stepanova et al., 2008). Ethylene application in cereals like rice roots leads to the endogenous accumulation of IAA. However, in *Yuc8/Rein7* mutants, the impact on auxin biosynthesis is minimal. Recently identified in rice, the zinc finger homeobox gene *OsZHD2* exhibits preferential expression in root tips and SAM. Overexpression of *OsZHD2* results in elongated seminal and lateral roots due to enhanced meristematic activity. *OsZHD2* stimulates root growth by promoting ethylene biosynthesis following auxin signaling (Yoon et al., 2020). Soil-surface rooting 1 (*SOR1*), a RING finger E3 ubiquitin ligase, regulates the stability of Aux/IAA proteins, thereby governing the root-specific ethylene response in rice (Chen et al., 2018). A unique auxin response factor (ARF), *TaARF4*, has been identified in wheat, showing promising results as a gene resource for controlling both root growth and plant height in wheat (Wang et al., 2019). **Figure 1B** describes different phytohormones function in root growth and development. NBD (2,5-norbornadiene) serves as an inhibitor of ethylene, a phytohormone crucially involved in various aspects of plant growth and development. In the context of adventitious root formation, ARR proteins likely engage in cytokinin signaling pathways, influencing root development. PAC, an inhibitor of gibberellin (GA), functions to suppress GA signaling during adventitious root formation, thus impacting the delicate balance of hormone interactions necessary for root initiation and growth. *WOX* genes encode transcription factors featuring the WUSCHEL-related homeobox domain. In the context of adventitious roots, *WOX* genes may play a regulatory role in the initiation and development of root primordia. In conclusion, these findings collectively underscore the multifaceted nature of hormonal regulation in wheat root development across various cereal crops, offering valuable insights for crop breeding and management strategies aiming for improved resilience and productivity.

### Phenotyping of the root system

**Figure 2:**
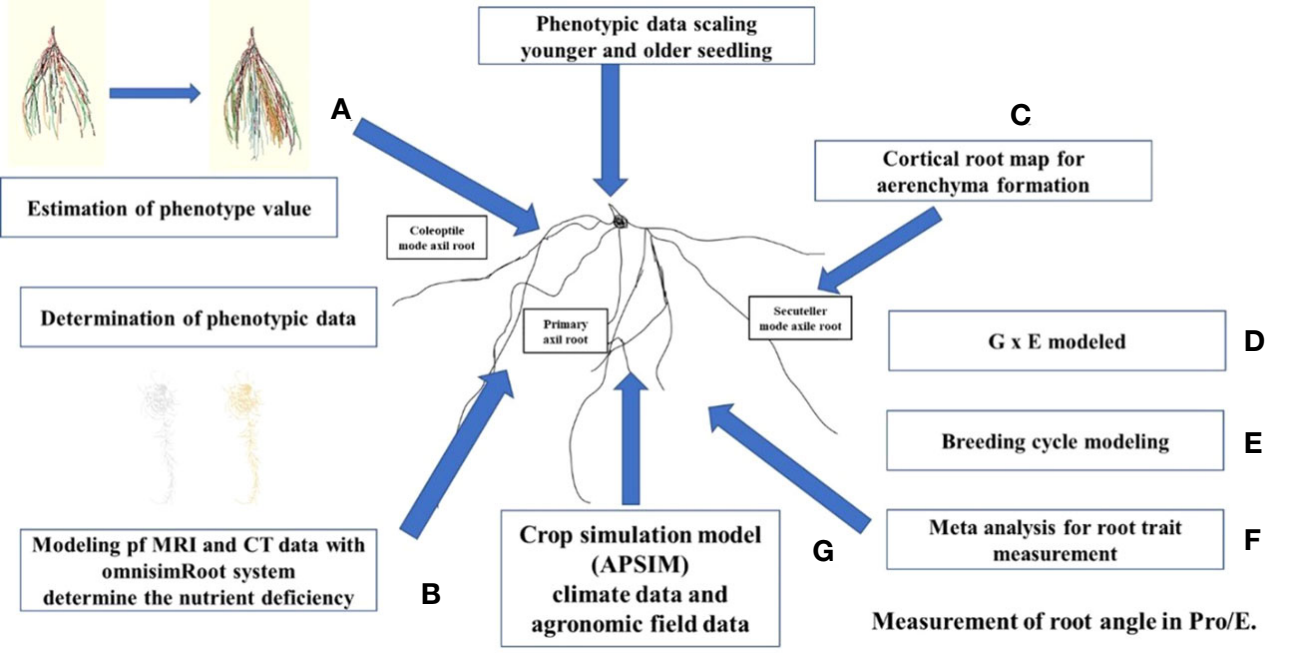
**(A)** Estimation of phenotype value. For example, determine the root branching and nutrient status with OpenSimRoot. **(B)** Determination of phenotypic data. Determine the variation among and within roots samples with function and structure models and predict the root angle using magnetic resonance imaging and computed tomography tools. Bayesian analysis may be used to classify the distribution of the core root and to increase the heritability of the deep root phenotype. **(C)** Phenotypical data scaling. Methods of phenotyping may be confined to early plant phases, because root structure and function of whole plants can be affected by cell senescence. **(D)** Modeled genotype x environment and management (G x E x M) helps to confirm the phenotypes using climate, soil and management practices for trait expression. **(E)** Breeding cycle helps to determine the trusted traits related to root structure and function. **(F)** Quantitatively evaluate the root traits. **(G)** Crop simulation model using climate data and field data for analysis.

The RSA not only structurally stabilizes plants but also provides photosynthate elements through water and nutrients and it protects leaves and reproductive organs from harmful elements and pathogens in the soil. Phenotyping facilitates the incorporation of root traits into root crops aiding in the development of new cultivars through prebreeding process (Tracy et al., 2020), and enabling their utilization in precision agricultural management (O’Toole and Bland, 1987). Modern phenotyping techniques provide automated, multi-sensor and non-destructive methods that use the whole plant, which can enable plants to be grown from seed and for their functional phenotypes to be determined, thereby accelerating the development of beneficial alleles. Additional benefits are expected from the introduction of field phenotyping into selection programs using soil root measurement technologies (Wasson et al., 2020). The distribution of phenotypes varies between roots and shoots based on conditions, i.e., dry, or moist environments (Chochois et al., 2012), availability of nitrogen (Gioia et al., 2015), availability of light (Walter and Nagel, 2006; Chochois et al., 2012) and soil strength (Chochois et al., 2012). Phenotyping systems can determine genetic variability in shoot division (Pfeifer et al., 2014), rooting (Barboza-Barquero et al., 2015), and how plant functional strategies shift according to soil humidity (Gioia et al., 2017). This throughput is ideal for the exploration of new characteristics, selection of breeding parents, and conducting QTL analysis. The progeny and elite germplasms can now be developed on a phenotyping platform with the capability for hundreds of rhizotrons (Tracy et al., 2020). Phenotyping platforms integrate measurements of water and nutrients in the soil and plant genotypes varying in water use efficiency (Nakhforoosh et al., 2016), salinity (Al-Tamimi et al., 2016) and nitrogen use efficiency (NUE) (Gioia et al., 2015). New space-time representations of plant dynamics provide an opportunity to understand the important characteristics for substantially reducing the energy requirements of soil stress tolerance (Arsova et al., 2020). An integrated approach for phenotyping RSA, illustrating the methods and functions of each instrument, is shown in **Figure 2**.

The dynamics of phenotypes are complex to determine because they require characterization in time and space dimensions. Advanced imaging technologies, such as magnetic resonance imaging (MRI), X-ray computed tomography (CT), and positron emission tomography (PET), can be applied for 4D phenotyping (Atkinson et al., 2019). These technologies measure root growth in various soil types and undisturbed soil cores (Metzner et al., 2015) with respect to water (Dietrich, 2018) and nutrients, including phosphorous and nitrogen (Flavel et al., 2014). Activation of meristematic activities in the development of an adventitious root (Jansen et al., 2014) and branch root (van Dusschoten et al., 2016) has been quantified by MRI, and the ^11^C root allocation has been observed in the same plants with PET and MRI imaging (Jahnke et al., 2009). Non-invasive root phenotyping techniques with electromagnetic radiation strengths (Wasson et al., 2020) are commonly used to monitor multiple tandem traits. This is achieved without damaging the plant and maintaining its viability (Jahnke et al., 2016). The shooting and rooting characteristics are combined in the correct seed at the stage of parent line growth. Following this step, different plant stages can be characterized that have desirable characteristics and these can be paired with an older plant stage with attractive properties to select features that ultimately enhance yield (Sadras and Richards, 2014).

Another method used to determine genetically acquired root traits is characterized by high reproducibility and low variability phenotypic screening. Controlling the surroundings for repeatable measurements is more successful in regions with volatile productivity (Richards et al., 2010). This is particularly true for the root system traits expressed in deep profile layers during later phases of plant development. The root system depth is poorly described at the stage of grain production in wheat under field condition (Watt et al., 2013), and quick measurements of the wheat canopy in the field, such as temperature (Lopes and Reynolds, 2010) and greenness (Li X. et al., 2019), are reliable rooting range predictors if the screening procedure is used under controlled conditions. Shovelomics techniques was published by Trachsel et al. (2011) and coring (Wasson et al., 2014) are invasive methods widely used for field-grown maize. These two approaches have a rhizotron-like output, as emphasized by MRI systems. Bayesian hierarchical nonlinear modeling helps determine root counts over the soil depth, which can be used as a single legacy characteristic (Wasson et al., 2017). Shovelomics can be used to quantify the traits of the washed root crown. The raw number, growth and angle of different species and soil conditions can be calculated (Trachsel et al., 2011). Digital imaging of root characteristics to estimate the shovelomics characteristics of roots along with multi-perspective imaging applications (Topp et al., 2016) can be used to standardize root measurements taken from different photographs of the same root crown while growing. Apparatuses have been developed for the washing of core fragments (Seethepalli et al., 2018) and the quantification of root length (Delory et al., 2017). Automatic cameras with image processing and positioning systems provide a modern mini-rhizotron imaging system (Vamerali et al., 2012). These have been used to establish genotypic variation to irrigation response (Ohashi et al., 2015), enhanced root development, and functional improvement among genotypes (Chen et al., 2019), as well as biotic root interactions, including the development of nodules to react with aboveground CO2 emissions. In another study, to collect 330,000 photos from 750 maize hybrids, 3,000 access tubes were installed for the acquisition of images, representing a paradigm of mini-rhizotron use in phenotyping (Tracy et al., 2020).

## Simulation models

Modeling is an emerging field of agricultural research that integrates scales and research systems (Marshall-Colon et al., 2017). Here, we present the available modeling approaches that support the phenotyping of roots, farming practices, and breeding for characteristics. OpenSimRoot (Postma et al., 2017) can be used to test the accuracies of root architectural model features. To help estimate the importance of phenotypes co-selected using technologies and their yield value across environments (Hoogenboom et al., 2004), OpenSimRoot may also use MRI and CT images of model roots from non-invasive scanning of soil (Daly et al., 2018). To study phenotypes and their importance under varying soil conditions, mechanistic models for plant growth and rhizosphere cycle have been constructed (Hoogenboom et al., 2004). Simulated responses to resource levels will substantially reduce the number of observations and test conditions required for phenotyping (Daly et al., 2018). Statistical models have also been used to link field root observations with features where simple linear correlations and normally distributed methods are insufficient (Schneider et al., 2017).

Phenotype relationships can be predicted by models within a specified plant age and phenology when desired (Zhao et al., 2017). Models in agronomic and breeding scales are helpful for the design of environmental and year-round activities. The importance of deep rooting in rain-fed systems has been modeled with over 100 years of climate data (Lilley and Kirkegaard, 2016). The combined selection of physiological traits within breeding cycles has also been modeled (Hammer et al., 2005). Recently, a study was performed to determine the root traits for breeding in wheat, rice and legumes based on phenotyping. The authors described 11 programs and modulation procedures for root trait improvement. Progress in this field has been successful but slow. These techniques combine several new germplasm alleles for target environments in parallel. Roots and shoots are simultaneously and non-destructively identified, and seed steps are automated with increasing comparisons between field and laboratory technologies. Simulation models that accept all phenotyping decisions have also been used. This century will witness progress toward phenotype production that will consider the dynamic characteristics of whole plant root systems, from single roots to dynamically manageable rhizosphere varieties on farms (Tracy et al., 2020).

## Molecular genetics of the root system (QTL, marker-traits association and SNP)

As roots are essential for capturing water and nutrients from the soil, it has been proposed that root exploitation characteristics, such as RSA, and improved nutrient uptake could lead to a new green revolution (Waines and Ehdaie, 2007). Breeders need to be able to select root elements suitable for effective soil and water nutrient acquisition. However, root selection is also difficult. QTLs analysis has been reported for several crops. Overlap in root-and-nutrient-capacity-related QTLs determination (Xie et al., 2017) has been shown in maize (Sinha et al., 2018), rice (Flintham and Gale, 1983) and wheat (Yuan et al., 2017), which indicates the useful potential of marker-assisted root feature breeding to improve resource efficiency and yields. Comprehending the genetic basis for root responses to nutrients availability is crucial for developing wheat varieties with an optimal root system adept at nutrient absorption within diverse soil environment. Previous studies have identified QTLs associated with wheat biomass and morphology (Laperche et al., 2006). However, a major challenge in pinpointing QTLs for root traits stems from the intricate nature of soil-root interactions, especially when analyzing multiple genotypes during QTL mapping. Root QTLs have primarily been identified through hydroponic or sand culture methods. While QTLs associated with wheat absorption and nutrient utilization in root products have been identified in hydroponics systems (Iannucci et al., 2017), their applicability in field trails for selecting root characteristics of soil-grown wheat plants remains uncertain. Ryan et al. (2015) searched for QTLs specific for root growth and uptake of nitrogen (N) and phosphorus (P), and used a recombinant inbred line (RIL) population for root morphology segregation, suggesting that Xiaoyan 54 responds more in terms of root growth relative to Jing 411 for N and P deficiencies. These findings showed the value of enhancing root biomass production for improving the efficiencies of N and P, regardless of being under low N, low P, or full-strength conditions. A total of 17 QTLs were identified for the root features investigated at 13 loci on 11 chromosomes. The findings showed that early strong growth enhances nitrogen and phosphate absorption in wheat (Ryan et al., 2015). P uptake QTLs (*qRDW.CK-2A, qSDW.LP-2A, qNUP.LP-2A, and qPUP.LP-2A*) have been reported in the seedling stage of P-deficient soil (Wang et al., 2016). Similar results have also been reported at the seedling and maturity stages with varying nitrogen and phosphorous availabilities (Wang et al., 2016).

A study has reported the identification of significant QTL for nodal root angle index (NRI) on chromosome 5B of wheat, utilizing 219 hexaploid winter wheat accessions. This locus is characterized by six SNP markers exhibiting strong linkage disequilibrium (LD), encompassing a region within the B-sub genome homoeolog of *VRN1*, a pivotal developmental gene in wheat and barley. The finding suggest that winter alleles consistently decreased root angle across all growth stages, under both greenhouse and field conditions. *VRN1* plays a dual role in regulating the timing and distribution of root growth throughout the wheat life cycle, with *VRN-B1* from the B-sub genome showing the most significant phenotypic impact (Voss-Fels et al., 2018b).

The identification of early-root growth quantitative trait loci (QTL) will improve the selection and efficiency of wheat varieties. Two major QTLs for seminal root morphology of wheat seedlings have been determined and these explained 68 and 59% of MRL and PRL phenotypical variation, respectively. Similarly, two major QTLs explained 30.5 and 24.5% of phenotypic lateral root length (LRL) and root tip number (RN) variation, respectively (Ren et al., 2012). Analysis of the QTLs distribution on chromosomes revealed two major clusters. One was named *qTaLRO-B1* and was positioned on the short arm of chromosome 2B. The second cluster was positioned on chromosome 6A. However, a detailed *qTaLRO-B1* study reported a negative allele domain and suggested primary root elongation impairment (Quraishi et al., 2011). Another study also supported the link with NUE (Ren et al., 2017). The importance of these three loci was further demonstrated by improved root growth and absorption of nutrients in wheat breeding. Xu et al. (2014) suggested that the *Rht-B1* (reduced height) gene is important for reducing plant height but not involved in root traits. Similarly, some studies found no significant effects of the *Rht-B1* gene on root traits (Schmidt et al., 2023). Alahmad et al. (2019) discussed seven GWAS-identified markers linked with root traits that were found based on cluster analysis on chromosome 6A, which recorded a higher linkage disequilibrium (LD) score (r^2^ = 0.67). In addition, the genetic predisposition to deep rooting was suggested to be better expressed with water restrictions, but in response to the supply of water, the root system showed plasticity in generating root growth. A QTL study established nine major QTL clusters on the 2A, 2B, 4B, 6A, 7A and 7B chromosomes in two-parent association mapping studies, which seemed most useful for reproductive applications (Zhang et al., 2021). Atkinson et al. (2015) identified 29 QTLs associated with wheat seedling root properties, and discovered one QTL specifically linked to root placement, impacting nitrogen uptake. This finding underscores a critical relationship among yield, nitrogen absorption and root characteristics. Richard et al. (2018) used a high-throughput screening for SRA in controlled circumstances, and the procedure was effectively applied in durum wheat, barley, and bread wheat. The method enabled independent, phenotypically based selection of cultivars centered on SRA (Robinson et al., 2018). Breeding lines were phenotyped to explore yield patterns (Laperche et al., 2006), and population mapping phenotyping was necessary for detecting QTLs (Ayalew et al., 2017). Some important information related to root development and elongation, as determined from QTL studies, is presented in **Supplementary Table S1**.

Considering the critical role of appropriate root phenotypes in crop yield, selection and breeding stand out as effective approaches for improving wheat varieties. Nevertheless, RSA is often omitted from breeding programs owing to its expensive and its time-intensive nature. Hence, there is considerable intrest in pinpointing RSA molecular markers for molecular breeding purpose (Rufo et al., 2020). Cattaneo and Hardtke (2017) identified the scarecrow and big brother genes as regulators of root growth and development. Marker and genome libraries compatible with the wheat reference genome IWGSCv1 have been developed using single-nucleotide polymorphism markers determined via next-generation sequencing. Gene annotations have been carried out for the identification of marker-trait associations (MTAs). A total of 63 MTAs were identified for seminal axes of root length (SAR), 24 for branching (BR), and 24 for total seminal root length (TSR). Additionally, putative proteins were uncovered for various diverse functions, i.e., MYB-TF for seminal axis root amino acid transporter for dry-root, and cinnamoyl-CoA reductase for root diameter regulation. Furthermore, these putative proteins were implicated in chymotrypsin, chalcone synthase, and aquaporin function (Beyer et al., 2019). Xu et al. (2021) utilized 390,136 SNP markers for GWAS in wheat, identifying a total of 484 SNPs associated with root traits. The SNP loci associated with NRT were the most detected, followed by those related to total root length (TRL), average root diameter (ARD), total root volume (TRV), total root area (TRA), and root dry weight (RDW). Xie et al. (2017) identified 38 QTLs associated with RSA. The occurrence of QTLs coincidence between root and yield traits suggests the presence of tightly linked RSA genes in wheat. Furthermore, the study identified three co-localized regions for root traits, including QTLs on chromosomes 1D, 4B, and 5D (Xie et al., 2017). Xu et al. (2021) identified five SNPs on chromosome 5B associated with RDW, name AX-111649489, AX-110419051, AX-109835270, AX-110550045, and AX-111547988 respectively. Beyer et al. (2019) used 20,881 SNP markers to identify different loci for root trait in wheat population. After analysis 59 MTAs markers were identified, 2 MTAs for root traits, 4 for TSR, 7 linked for SAR, 24 were for BR, 20 were associated with RD, and 8 for linked with RDM. Similar this study also reported that the two genes, TraesCS5B01G487600 and TraesCS5B01G488000, encoding putative 12-oxophytodienoate reductase-like protein and syntax in showed significant root function in the wheat. Rufo et al. (2020) utilized 170 bread wheat landraces from 24 Mediterranean countries to pinpoint molecular markers linked to RSA and attributed traits. The findings revealed 135 marker-trait associations through GWAS, elucidating 6-15% of the phenotypic variances for root-related traits Notably, fifteen QTL hotspots emerged as significant regulators of root trait variation, 31 candidate genes associated with RSA traits, root development, and abiotic stress tolerance, primarily drought. Furthermore, co-localization of root-related traits, with only four of the fifteen QTL hotspots having been previously documented (Rufo et al., 2020). Assessing its efficacy in tetraploid wheat breeding, an F6 hybrid breeding population resulting from numerous crosses using *T. durum* cultivars, as well as wild and cultivated emmer wheat accessions. Genetic map constructed using combine linkage and association mapping strategy from 1345 SNP markers. The results suggested that six QTLs were found: two for coleoptile length, one for heading date, one for anthocyanin accumulation, and two for osmotic stress tolerance (Hussain et al., 2021).

## Genetic engineering to improve root traits in wheat

The available genomic tools for genome editing aid in identifying the gene of interest (GOI), functionally classifying genetic materials, and transferring the GOI to leverage its regulatory activities for enhancing the wheat genome. These tools can subsequently be applied to implement targeted genes under variable environments where wheat cultivation occurs globally (Borisjuk et al., 2019). The common wheat genome contains more than 128,000 genes (Bhalla et al., 2017), with more than 80% of the genome comprising repetitive DNA sequences (Appels et al., 2018). A total of 107,891 high-confidence genes with more than 85% replicated DNA sequences have been identified, represented as triple redundancies by the hexaploid genome (Shrawat and Armstrong, 2018). The wheat genome has evolved in complexity with relative recalcitrance to *in vitro* culture and recovery in most varieties (Vasil et al., 1992).

Zhang et al. (2014) produced transgenic wheat plants overexpressing phosphoenol/pyruvate carboxylase (*PEPC*) and pyruvate, orthophosphate dikinase (*PPDK*), both separately and simultaneously, within the same transgenic background. The results suggested that the PEPC- gene enhanced drought tolerance and boosted grain yield in transgenic wheat (*Triticum aestivum* L.) plants (Qin et al., 2016). A maize gene TF *Dof1* that is essential for *PEPC* upregulation was transferred to wheat by transformation using Agrobacterium (Peña et al., 2017), which encouraged root growth and upregulation in the transgenic wheat roots of both the nitrate and phosphate transports, resulting in increased nutrient uptake.

The overexpression of *TaNAC2-5A*, another TFs present in wheat, plays a role in nitrogen signaling, enhancing root growth, and improving the root’s ability to absorb soil nitrate (He et al., 2015). Wheat grains primarily acquire nitrogen through two main pathways: uptake from the canopy (leaves and stems) and absorption from the soil roots. Apart from being a crucial plant nutrient in crop production, nitrogen accumulation also significantly influence the composition of wheat grain storage protein (Zörb et al., 2018). Given their specific involvement in root gravitropic angle (RGA) regulation without influencing other morphological traits, *DRO1*-family genes and the *EGT2* gene present promising targets for GE to enhance tolerance to diverse abiotic stressors. Extending this knowledge to other cereal crops and evaluating their responses to various stress factors would be intriguing (Kořínková et al., 2022). Another study also used CRISPR/Cas9-based system, denoted LR-1 and LR-2 constructs containing dual guided RNAs, was employed for targeted mutagenesis of *TaRPK1* genes in *Triticum aestivum*. These constructs were introduced via Agrobacterium to modify root architecture, consequently affecting yield. Furthermore, the results suggested that the transgenic lines exhibited substantial changes in morphology and RSA, with a notable rise in the number of effective tillers, grain weight, root length, root depth, root volume, and root surface area, alongside a decrease in root diameter, root angle, and spike length, relative to wild plants. The significant increase in tillers and total grain weight indicates that edited lines enhanced grain production by reducing spike length (Rahim et al., 2024).

## Prospects

Roots are an important plant organ that determine the absorption of water, which is important for plant survival during a drought, as well as of nutrients resulting from the activity of soil microbes. Therefore, the design and development of new root system ideotypes should be a priority for wheat breeding in the future. Scientists who wish to research the characteristics of wheat roots should be mindful that this will require the investment of considerable time and effort. Many breeding systems have conventional root parameters that have not yet been examined. For wheat adaptation, root features that affect characteristics such as drought tolerance, element toxicity, disease management, and waterlogging should be assessed. Therefore, when analyzing root traits that affect selection and reproduction, target climate, rainfall and water stress patterns should all be considered. Wheat breeders should search for root characteristics to improve plant growth and plant production to create improved wheat varieties. With nutrient shortages, RSA can be modified to ensure optimal capture of nutrients, plant survival and crop yield. Through the manipulation of recently established nutrient carriers and of quantitative traits for root angle, a reproduction program for nutrient-capturing crop lines can be improved. To optimize and refine crop production, the functional similarities and differences shared by these advantageous roots must be understood.

Wheat breeding has led to the substantial alteration of many roots functional traits due to the group selection of these traits. The SRs features have been compacted at the cost of individual fitness to increase population growth. Smaller and deeper root systems with simpler structures and fewer fractal dimensions have resulted in shorter SRs with reduced growth angles that perform better under agricultural conditions. The variation in Mediterranean wheat landraces with the newly established QTL hotspots shows that land breeds have reliable rooting characteristics that could be introduced in modified phenotypes through marker-assisted breeding (Rufo et al., 2020). The reports of extreme genotypes can be used as a basis for the creation of new attempts to map the corresponding features among the different characteristic associations. To date, retrospective studies of trait-based root enhancement show significant potential, but new innovations and technologies should be incorporated into the rhizosphere system along with agronomy and breeding tools for quicker response. Integrated programs such as NUE associated with biological nitrification inhibitors from root exudates are making rhizosphere phenotypes accessible (O’Sullivan et al., 2016) and enhancing water availability management of the root branch architecture (Bao et al., 2014). A study of characteristics selected for water-limited cereals has also showed that the time between phenotype conception and proof of germplasm definition for breeders was too long to satisfy food security requirements (Hall and Richards, 2013). In the future, phenotyping technology could be used to quantify the crop-integrated phenotype through management activities, soil inputs, and new breeding technologies. Rhizospheres may be combined with data from highly transmitted canopies for phenotyping, which is very useful for distinguishing seedlings and mature stages in wheat crops (Bai et al., 2016). Additional pattern recognition software could also be used to recognize mixed phenotypes and rhizospheres correlated with advantageous selection and management characteristics for new algorithms (Mohanty et al., 2016). Attempts have been made to use phenotyping, robotics, and computational technology to improve crop management synergy (Fiorani and Schurr, 2013). Genome technologies are also advancing through epigenetics (Quadrana and Colot, 2016) and with microbiome plants being viewed as halobionts (Vandenkoornhuyse et al., 2015). A recent study suggested a toolkit for rapid root system modification using single plant selection (SPS), allowing for selection of plants with extreme root phenotypes without destroying them. This method was utilized to develop introgression lines with varied root structures but similar plant height and flowering time to the original parent. These elite wheat lines with modified root traits offer valuable resources for evaluating the impact of root characteristics on yield enhancement across diverse environments and production systems within a specific genetic context. The SPS approach presents a valuable framework for researchers and breeders aiming to enhance root systems in future crop varieties (Rambla et al., 2022).

## Conclusion

In conclusion, the pivotal role of roots in facilitating a plant’s uptake of water and nutrients, crucial for its survival in droughts and overall well-being, cannot be overstated. It is paramount to prioritize the development of novel root systems, particularly in wheat breeding, to bolster traits linked to drought tolerance, nutrient absorption, disease resistance, and tolerance to waterlogging. Wheat breeding initiatives have significantly altered root functional traits, emphasizing the selection of specific traits that promote population growth but may compromise individual fitness. Nevertheless, valuable attributes observed in landraces and extreme genotypes present promising avenues for marker-assisted breeding and trait improvement. Advancing research on roots necessitates the integration of cutting-edge technologies such as phenotyping, robotics, computational tools, and genomic advancements. These tools offer opportunities for deeper root system comprehension, hastened gene discovery, and the creation of wheat varieties resilient and productive across varied conditions. Collaboration, innovation, and a comprehensive understanding of root systems will be pivotal in shaping the future of wheat breeding and crop production.

## Author contributions

FA: Supervision, Visualization, Writing – original draft, Writing – review & editing. AA: Investigation, Writing – original draft, Writing – review & editing. SA: Conceptualization, Methodology, Writing – review & editing. SB: Project administration, Writing – review & editing.
